# In-hospital survival paradox in patients with sleep apnea—A nation-wide nested case-control study

**DOI:** 10.1371/journal.pone.0271004

**Published:** 2022-07-21

**Authors:** Maurice Moser, Florent Baty, Martin H. Brutsche, Otto D. Schoch

**Affiliations:** Lung Center, Cantonal Hospital St. Gallen, St. Gallen, Switzerland; Sapienza University of Rome, ITALY

## Abstract

**Background:**

Sleep apnea (SA) is a prevalent disorder characterized by recurrent events of nocturnal apnea originating from obstructive and/or central mechanisms. SA disrupts normal sleep and can lead to a series of complications when left untreated. SA results in intermittent hypoxia which has an impact on the cardio- and cerebrovascular system. Hospitalized patients with SA typically have a greater burden of comorbidity, a longer length of hospital stay, but may show an improvement of in-hospital mortality compared to patients without diagnosed SA. The reason for this survival benefit is controversial and we aimed to clarify this protective effect in the light of predictive factors including SA-associated comorbidities using a nation-wide hospitalization database.

**Methods and findings:**

Data were extracted from a nation-wide hospitalization database provided by the Swiss Federal Office for Statistics. Hospitalized patients with a SA co-diagnosis were extracted from the database together with a 1:1-matched control population without SA. Overall, 212’581 patients with SA were hospitalized in Switzerland between 2002 and 2018. Compared to the controls, SA cases had a longer median length of hospital stay (7 days; 95% CI: 3 to 15 vs. 4 days; 95% CI: 2 to 10) (*p* < 0.001) and a higher median number of comorbidities (8 comorbidities; IQR: 5 to 11 vs. 3 comorbidities; IQR: 1 to 6) (*p* < 0.001). The risk of in-hospital mortality was lower in the SA cases compared to the controls (OR: 0.73; 95% CI: 0.7 to 0.76; *p* < 0.001). SA was associated with a survival benefit in hospitalizations related to 28 of 47 conditions with the highest rate of in-hospital death. Sixty-three comorbidities were significantly over-represented in SA cases among which obesity, hypertension and anatomic nasal deviations were associated with a significant decrease of in-hospital mortality.

**Conclusions:**

Compared to matched controls, SA was associated with significant and relevant inpatient survival benefit in a number of most deadly conditions. Within SA-patients, associated comorbidities mostly correlated with a poorer prognosis, whereas obesity and hypertension were associated with an improved in-hospital mortality.

## Introduction

Sleep apnea (SA) is a prevalent condition characterized by recurrent events of nocturnal apnea resulting in oxygen desaturations, activation of the vegetative nervous system and fragmented sleep [1, 2]. Obstructive and central sleep apnea are two prominent subtypes of sleep apnea. An untreated SA can increase the risk of stroke, cardiovascular diseases, diabetes, car accidents and depression [3–8]. Sleep deprivation associated with OSA may also induce neurodegeneration [9, 10].

SA is usually diagnosed by polysomnography (PSG) or respiratory polygraphy and can be further characterized by advanced endoscopy techniques [11].

SA is often accompanied by different comorbidities which can be categorized as etiological, consequential or concomitant. Previous review articles have systematically summarized evidence demonstrating relevant associations between SA and conditions including, endocrine, cardiovascular, metabolic, hematological, renal, respiratory, gastrointestinal, neurological, ophthalmological and psychiatric diseases [8, 12–21]. SA is associated with higher body mass index, diabetes, older age and male gender [22]. Furthermore, SA-associated intermittent hypoxia leads to reactive oxygen production, which may trigger chronic inflammation frequently associated with other inflammation disorders such as metabolic syndrome or nasal chronic inflammation [23].

The outcome of hospitalized patients with SA has only been scarcely investigated and is still controversial [24, 25]. Patients with SA typically have a greater burden of comordities, use more hospital resources [26] but, for certain conditions (including myocardial infarction, pneumonia, pulmonary embolism), have a lower inpatient mortality [25, 27–29].

The aim of the current study was, thus, to investigate and decipher the survival benefit in hospitalized patients with sleep-apnea in the light of various predictive factors including SA-associated comorbidities using a nation-wide hospitalization database.

## Materials and methods

### Swiss hospitalization database

Inpatient data were extracted from a hospitalization database provided by the Swiss Federal Statistical Office. The database offers a nation-wide coverage of all hospitalizations in Switzerland. Patient information was fully anonymized and no written consent was required. No ethical approval was required for the current retrospective study. All diagnoses were coded using the German modification of the International Classification of Disease version 10 (ICD-10-GM). The list included one main diagnosis and up to 50 additional co-diagnoses.

The database included 24’239’724 hospitalization entries in the period between 2002 and 2018 (17 years). Every patient had a unique anonymous identifier. Information included the year and month of hospitalization, the patient’s age and gender, the length of hospital stay (LOS) and in-hospital mortality, as well as the patient’s region of residence and the canton of the institution.

The data set was imported into an SQL database (SQLite version 3.31.1) and interfaced with the R statistical software using the dedicated package RSQLite.

### Sleep apnea cases and nested case-control design

**Fig 1:**
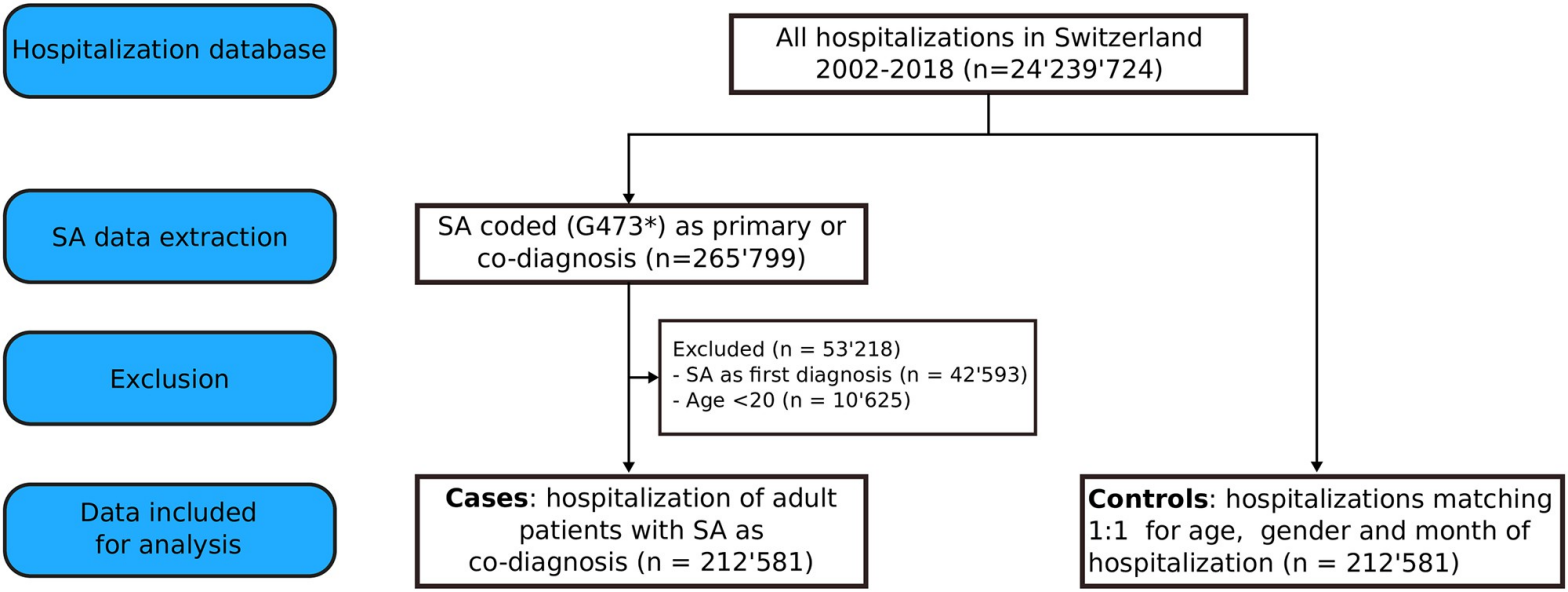
Flow diagram of the study protocol. The study follows a nested case-control design. The cases correspond to the hospitalizations of adults patients with a SA co-diagnosis. The controls correspond to hospitalizations without coded SA in patients matching the cases 1:1 for age, gender and month of hospitalization.

Fig 1 depicts a flow diagram of the current study protocol, in line with the STROBE guidelines [30]. All adult sleep apnea cases (≥ 20 years old), i.e. patients hospitalized with a co-diagnosis of SA (ICD-10-GM codes G473*), were extracted from the database. All hospitalizations with a primary diagnosis of SA (i.e. initial SA assessment using PSG) were excluded from the current analysis. Since 2012, overnight PSG assessments have been performed in an outpatient setting and are not recorded in the Swiss hospitalization database. SA sub-codes included obstructive and central sleep apnea, as well as unspecified / other types of SA.

A control population without co-diagnosis of SA was obtained from the database using a random extraction procedure. This 1:1 nested-control population was matching the SA cases for age, gender and month of hospitalization.

### Sleep apnea-associated comorbidities

Comorbidities associated with SA were defined as any ICD-10-GM code used concomitantly to SA. The comorbidities of interest were the ones over-represented in the SA cases compared to the matching controls.

### Statistical considerations

Patient’s baseline characteristics were summarized using descriptive statistics. Fisher’s exact test for count data, or alternately conditional logistic regression, was used to identify comorbidities significantly over-represented in the SA cases. Results were reported as odds-ratio together with 95% confidence intervals and associated *p*-values. Comorbidities of interest were selected based on their significance levels (adjusted *p*-value < 0.05) and the sign of the odds ratio (> 1).

The presence-absence of comorbidity in each SA case was compiled into a table of 0/1s and the correlations among over-represented comorbidities were investigated using principal component analysis (PCA). A vector fitting procedure was used in order to facilitate the interpretation of the PCA results with the help of external explanatory variables.

The effect of selected comorbidities on in-hospital mortality was investigated using conditional logistic regression. The effect of the comorbidities was reported using adjusted odds-ratio and associated 95% confidence intervals. The interaction terms between comorbidities and SA were also reported together with their standard error.

The impact of SA on the in-hospital mortality of patients hospitalized for prevalent severe conditions was further investigated. Prevalent severe conditions were defined as primary diagnoses with a prevalence higher than 1/10’000 an in-hospital mortality greater than 10%.

All analyses were done using the R statistical software (v. 4.0.4) including the dedicated packages ADE4, vegan, comorbidity and ICD10gm.

## Results

### Characteristics of SA hospitalizations in Switzerland

**Fig 2:**
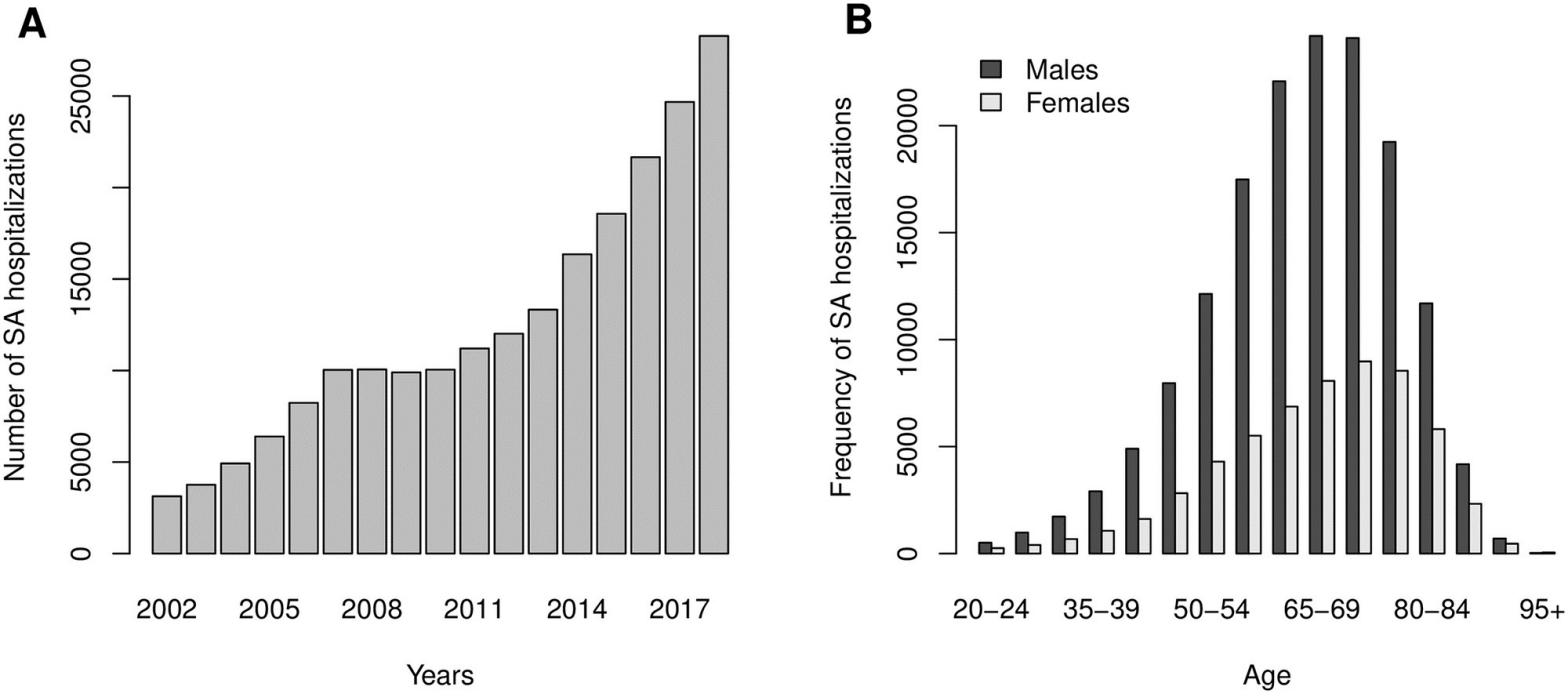
Characteristics of SA hospitalizations in Switzerland. The evolution of the annual number of SA hospitalizations between 2002 and 2018 is shown in panel A. The number of hospitalizations with a co-diagnosis of SA increases steadily over the years. The age distribution per gender is depicted in panel B.

The number of hospitalizations coded with a co-diagnosis of SA between 2002 and 2018 was 212’581 (0.9% of all hospitalizations) corresponding to 108’242 unique patients. The evolution of the number of hospitalizations with a co-diagnosis of SA showed a steady increase from less than 5’000 cases in 2002 up to more than 25’000 cases in 2018 (Fig 2, panel A). Overall, 73 percent of patients were males. The age distribution of SA cases in both genders is shown in Fig 2 (panel B). The mode of the age distribution was 65-69 years for males and 70-74 years for females.

The ICD-10-GM code for SA (G473) was subdivided into different sub-categories including obstructive SA (50% of cases), central SA (3% of cases), and other, unspecified or not otherwise specified SA (47% of cases).

Table 1 provides a comparative overview of the baseline characteristics of SA cases compared to matched controls. The median number of comorbidities was significantly higher in the SA cases (8; IQR: 5 to 11) compared to the controls (3; IQR: 1 to 6) (*p* < 0.001). SA cases had a higher median Charlson’s comorbidity index (1; IQR: 0 to 2) compared to the controls (0; IQR: 0 to 1) (*p* < 0.001). The median LOS was significantly longer in the SA cases (7 days; IQR: 3 to 15) than in the controls (4 days; IQR: 2 to 10) (*p* < 0.001). The in-hospital mortality was significantly lower in SA cases (1.8%; 95% CI: 1.8 to 1.9%) compared to the controls (2.5%; 95% CI: 2.4 to 2.6%). The associated odds-ratio for in-hospital mortality was 0.73 (95% CI: 0.70 to 0.76; *p* < 0.001). The survival benefit of SA was found in all SA sub-types except central sleep apnea where the in-hospital mortality did not significantly differ from the controls.

**Table 1:** Baseline characteristics of hospitalized cases with SA compared to age and sex-matched controls without diagnosis of SA (1:1 nested case-control design).

	SA-cases	Controls
Number of hospitalizations (*n*)	212’581	212’581
Age (mode)	65-69	65-69
Unique patients (*n*)	108’242	200’705
Median length of stay in days (IQR)	7 (3 to 15)	4 (2 to 10)
% in-hospital mortality (95% CI)	1.83 (1.77 to 1.89)	2.49 (2.43 to 2.56)
Median number of comorbidities (IQR)	8 (5 to 11)	3 (1 to 6)
Median Charlson’s comorbidity index (IQR)	1 (0 to 2)	0 (0 to 1)
Most prevalent reasons for hospitalization	I50: Heart failure (4.1%) J44: Chronic obstructive pulmonary disease (4.09%) E66: Obesity (3.86%)	I25: Ischaemic heart disease (2.34%) K40: Inguinal hernia (2.21%) M17: Gonarthrosis (2.17%)
Most prevalent reasons for in-hospital mortality	I50: Heart failure (9.86%) A41: Sepsis (7.7%) J96: Respiratory failure (6.2%)	C34: Lung cancer (6.13%) A41: Sepsis (5.08%) I21: Myocardial infarction (3.72%)

The most prevalent conditions for hospitalization were heart failure (4.1%), chronic obstructive pulmonary disease (4.1%) and obesity (3.9%) in the SA cases and chronic ischemic heart disease (2.3%), inguinal hernia (2.2%) and osteoarthritis of knee (2.2%) in the matched controls.

The most prevalent conditions associated with high in-hospital mortality were heart failure (9.9%), sepsis (7.7%) and respiratory failure (6.2%) in the SA cases and lung cancer (6.1%), sepsis (5.1%) and acute myocardial infarction (3.7%) in the matched controls.

## In-hospital mortality

### In-hospital mortality and SA comorbidome

Overall, a total of 10’407 comorbidities were coded in association with SA. Among these comorbidities, 63 were significantly overrepresented compared to the matched control population (Table 2). SA-associated comorbidities included bacterial infections (B96), blood disorders (D50, E03, E11, E55, E78, E79, E87), hypokalaemia (E876), obesity (E66), psychological disorders (F171, F329), neurologic diseases (G25, G63), heart disease and hypertension (I10, I11, I25, I27, I48, I50), respiratory tract diseases (J44, J96), anatomic deviations in the respiratory tract (J34), asthma (J45), gastroesophageal reflux disease (K21), kidney diseases (N08, N18), cardiac devices (Z95) and respiratory devices (Z99).

**Table 2:** List of comorbidities significantly over-represented in SA compared to the matched-control population. The simplified ICD-10-GM codes including 2 digits are presented together with a short description and the associated odds-ratios.

ICD-10-GM code	Description	Odds-ratio (95% CI)
Z99	Dependence (long-term) on enabling machines and devices	10.3 (95% CI: 9.7 to 11)
E66	Obesity	9.9 (95% CI: 9.6 to 10.1)
J96	Respiratory failure, not elsewhere classified	6.1 (95% CI: 5.9 to 6.3)
I27	Other pulmonary heart diseases	5.8 (95% CI: 5.6 to 6.1)
J34	Other disorders of nose and nasal sinuses	5 (95% CI: 4.7 to 5.4)
G25	Other extrapyramidal and movement disorders	5 (95% CI: 4.7 to 5.3)
N08	Glomerular disorders in diseases classified elsewhere	4.6 (95% CI: 4.3 to 4.8)
J45	Asthma	4.2 (95% CI: 4 to 4.4)
J44	Other chronic obstructive pulmonary disease	4 (95% CI: 3.9 to 4.1)
E11	Type 2 diabetes mellitus	3.8 (95% CI: 3.7 to 3.9)
G63	Polyneuropathy in diseases classified elsewhere	3.7 (95% CI: 3.5 to 3.9)
E79	Disorders of purine and pyrimidine metabolism	3.7 (95% CI: 3.5 to 4)
I11	Hypertensive heart disease	3.6 (95% CI: 3.6 to 3.7)
I50	Heart failure	3.4 (95% CI: 3.4 to 3.5)
N18	Chronic kidney disease	3.1 (95% CI: 3.1 to 3.2)
E78	Disorders of lipoprotein metabolism and other lipidaemias	3.1 (95% CI: 3.1 to 3.2)
E03	Other hypothyroidism	3 (95% CI: 2.9 to 3.1)
E55	Vitamin D deficiency	3 (95% CI: 2.8 to 3.1)
I48	Atrial fibrillation and flutter	2.8 (95% CI: 2.7 to 2.8)
K21	Gastro-oesophageal reflux disease	2.7 (95% CI: 2.7 to 2.8)
I10	Essential (primary) hypertension	2.6 (95% CI: 2.6 to 2.7)
Y57	Side effects of drugs and medicaments in therapeutic usage	2.6 (95% CI: 2.5 to 2.7)
Z92	Personal history of medical treatment	2.3 (95% CI: 2.2 to 2.3)
Z95	Presence of cardiac and vascular implants and grafts	2.3 (95% CI: 2.2 to 2.3)
F32	Depressive episode	2.3 (95% CI: 2.2 to 2.3)
B96	Other specified bacterial agents as the cause of diseases	2.2 (95% CI: 2.2 to 2.3)
Z86	Personal history of certain other diseases	2.2 (95% CI: 2.2 to 2.3)
E87	Other disorders of fluid, electrolyte and acid-base balance	2.2 (95% CI: 2.2 to 2.3)
I25	Chronic ischaemic heart disease	2 (95% CI: 2 to 2)
F17	Mental and behavioural disorders due to use of tobacco	2 (95% CI: 1.9 to 2)
Y84	Surgical or other medical procedures as the cause of complication	1.9 (95% CI: 1.8 to 1.9)

**Fig 3:**
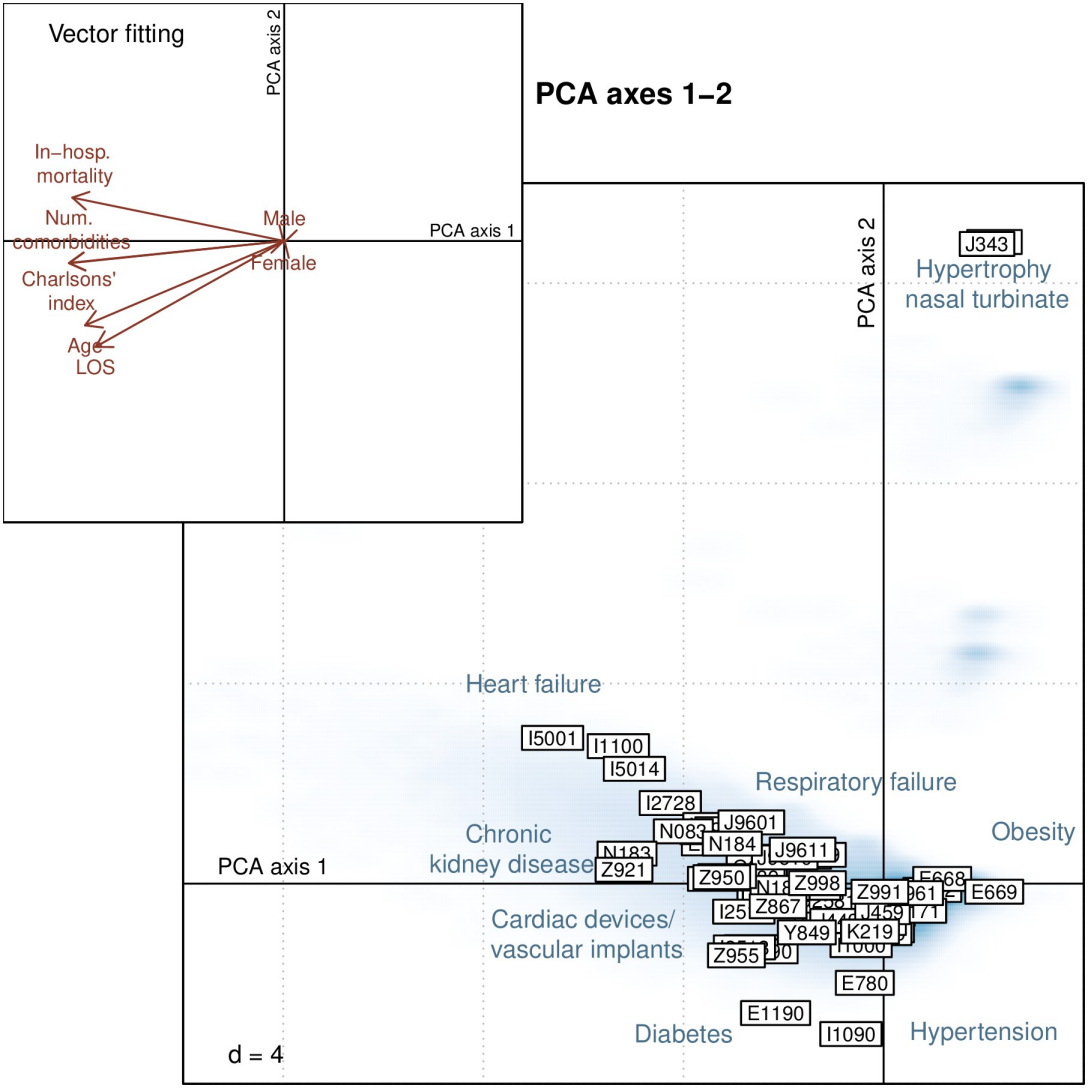
Principal component analysis (PCA) biplot of comorbidities associated with sleep apnea. SA hospitalization cases are reported in smoothed blue density areas, whereas comorbidities (ICD-10-GM codes) are depicted by framed labels. Comorbidities lying in the same direction are correlated. Comorbidities lying far away from the center of the plot are the most influential comorbidities. As a help for interpretation, the upper left inset represents external explanatory variables fitted to the PCA plot. The association between comorbidities and explanatory variables is given by the direction of the arrows.

The correlations among SA-associated comorbidities and outcome are summarized in the PCA biplot shown in Fig 3. A gradient of comorbidities can be observed along the first PCA axis ranging from obesity (E668, E669) and hypertension (I10) on the right side (high scores) to heart failure (I50), chronic kidney diseases (N183, N184), respiratory failure (J96) and presence of a cardiac device / vascular implant (Z95) on the left side (low scores). Comorbidities with the lowest scores on the first PCA axis were associated with a poor prognosis (i.e. higher in-hospital mortality, longer LOS, higher number of comorbidities and worse Charlson’s comorbidity index) and also correlated with age. The second PCA axis discriminated patients with anatomic deviations of the respiratory tract (J342, J343), which represent a cluster of comorbidities independent from the other set of comorbidities, more frequently present in males.

Clear associations between comorbidities and in-hospital mortality were found. Respiratory and heart failures were mostly associated with a high-risk of in-hospital mortality, whereas obesity and hypertension had a protective effect. The observed protective effect of obesity (E66) and essential primary hypertension (I10) was further scrutinized using logistic regression. A significant inpatient survival benefit was associated with obesity in both SA cases and matched controls (adjusted OR: 0.987 (95% CI: 0.983 to 0.990; *p* < 0.001). A significant interaction effect between SA and obesity was found (*b* = 0.0095, SE = 0.002; *p* < 0.001). The positive sign of the interaction term indicated that the protective effect of obesity was significantly smaller in the SA compared to the controls. A similar significant interaction effect was found with other metabolic comorbidities including lipidemia (E78) and vitamin D deficiency (E55). Regarding hypertension, a protective effect was also found in both SA and controls. A significant negative interaction effect indicated that the protective effect was larger in the SA compared to the controls (*b* = -0.0027, SE = 0.001; *p* < 0.001).

### In-hospital mortality of SA in severe conditions

**Fig 4:**
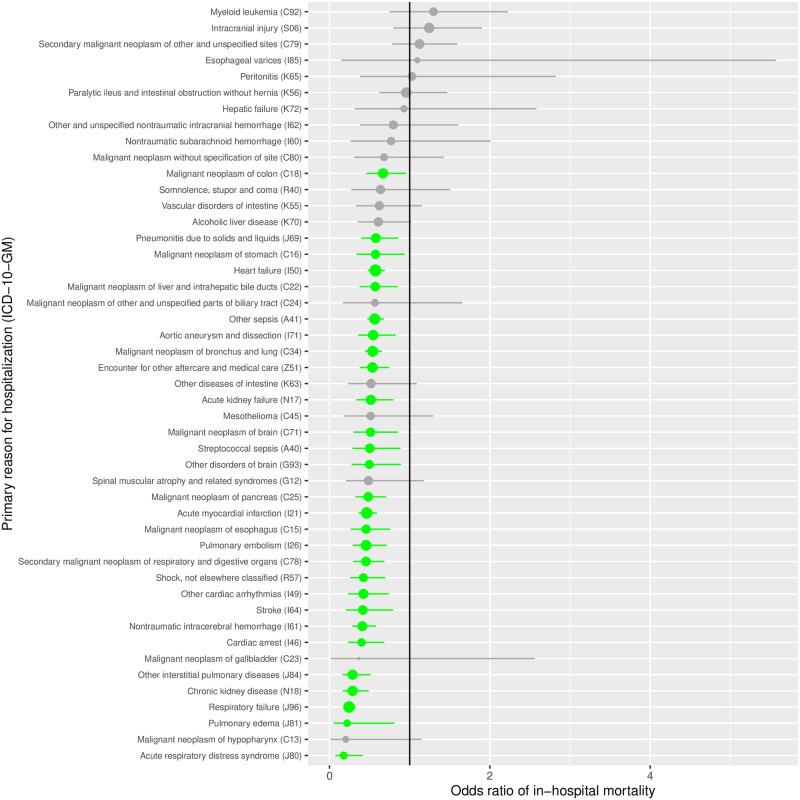
Survival benefit of coded sleep apnea in patients hospitalized with 47 conditions with highest in-hospital mortality. The odds-ratios (and 95% confidence intervals) comparing the in-hospital mortality between SA cases and matched controls are displayed using a forest plot. The estimates are represented by dots whose size is proportional to the prevalence of the condition. Comorbidities significantly associated with a lower rate of in-hospital mortality are shown in green. Comorbidities not significantly associated with in-hospital mortality are shown in gray. The simplified ICD-10-GM codes including 2 digits are presented.

A list of 47 prevalent diseases with the highest rates of in-hospital mortality was extracted from the hospitalization database. The effect of a co-diagnosis of SA (compared to non-SA) on in-hospital mortality is depicted using a forest plot (Fig 4). In hospitalizations due to 28 out 47 most deadly conditions, SA diagnosis was associated with a significant decrease of in-hospital mortality. Furthermore, in none of 47 most deadly diseases, SA co-diagnosis was associated with an increased in-hospital mortality. An SA co-diagnosis was associated with a strong in-hospital benefit in critical cardiopulmonary disease states including respiratory failure, pulmonary edema, interstitial pulmonary disease, acute respiratory distress syndrome, pulmonary embolism as well as cardiac arrest, arrhythmia, acute myocardial infarction, heart failure. SA co-diagnosis was also associated with an in-hospital survival benefit in association with various neoplasms (esophagus, pancreas, brain, lung, liver, stomach, colon). Other in-hospital survival benefit was found in association with conditions including sepsis, chronic kidney diseases and aortic aneurysm.

## Discussion

In the current study, the observed inpatient survival benefit of SA was analyzed in the light of predictive factors including SA-associated comorbidities and primary reasons for hospitalization.

SA is a condition with a high prevalence, especially in the elderly population. Considering the increase in the average age of the world population, it is essential to diagnose and treat this disease in elderly patients [31]. SA was associated with a longer hospital stay and an increased burden of comorbidities. Among frequent complications, SA is strongly associated with cardiac arrhythmias and atrial fibrillation indicating fluctuations of SA-related autonomic nervous system [32]. Altered cognitive performance are also frequently observed [33]. Despite of these unfavorable pre-conditions, we found a remarkable decrease of 28% for in-hospital mortality in patients with SA (1.8%) compared to matched controls (2.5%).

Our results are in line with other studies in patients with SA. Longer LOS and a larger burden of comorbidities explains the observed over use of hospital resources [26]. The significant reduction of in-hospital mortality for patients coded with SA has been previously described for several conditions. Lindenauer and colleagues were among the first to describe an inpatient survival benefit in SA patients hospitalized with pneumonia [28]. Another early study found an unexpected decrease of in-hospital mortality in patients with SA admitted in the intensive care unit [34]. These findings were confirmed independently in a more recent study [35]. Similarly, other recent publications involving patients hospitalized for myocardial infarction [25, 27], pulmonary embolism [29] and critically ill patients [36], showed a decrease of in-hospital mortality associated with diagnosed SA. The mechanisms underlying these findings are incompletely understood. Some authors have postulated that SA patients are younger and tend to have a significantly larger comorbidity profile [25, 28, 37, 38], but are more likely to be adequately treated than the controls. Furthermore there is an increased chance that patients with SA will be taken to referral centers which may have a better position to handle more complex patients [28]. It is well known that SA is associated with obesity which in turn, is associated with a better outcome compared to non-obese patients with an established cardiovascular disease. This phenomenon is known as the “obesity paradox” [39]. Obese patients may show earlier cardiovascular symptoms possibly accompanied with optimized medical treatments [40]. Our data show that obesity has a protective effect in both SA cases and matched controls. However the effect is attenuated in the SA-cases compared to the controls, indicating that SA and not obesity might be the predominant explanation for the survival benefit.

Another hypothesis postulates that SA-related hypoxic preconditioning might be beneficial [25, 41–44]. Pathophysiologically, the oxidative stress associated with the intermittent hypoxia of sleep apnea may trigger cardio- and cerebro-protective mechanisms, a phenomenon which had been reviewed in detail recently [45]. Some studies showed that intermittent hypoxia is associated with less severe outcome in myocardial infarction [46, 47]. Ozeke and colleagues further confirmed the role played by chronic intermittent hypoxia due to SA, in the morbidity-mortality paradox of obesity [48].

Our study has several limitations. The data analyses are based on hospitalized cases with a coded SA, a condition which might still be underdiagnosed or underreported in patients hospitalized for other reasons. The coding skills of the healthcare worker may impact on the completeness of the identified SA cases. SA was possibly coded only if the disease played a clinically relevant role. PSG parameters and other detailed SA-related diagnostic information were not available in the current hospitalization database. In addition, the current study is in essence retrospective and no causality can be inferred.

## Conclusion

Our study confirms that SA is significantly associated with a clinically meaningful inpatient survival benefit in at least 28 of the 47 deadliest conditions. SA-associated comorbidities were frequent and significantly influenced patient centered outcomes.

## Abbreviations

AHI: Apnea-hypopnea index
ICD-10-GM: International classification of disease, revision 10, German modification
LOS: Length of hospital stay
PCA: Principal component analysis
PSG: Polysomnography
SA: Sleep apnea
SQL: Structured query language

## References

[1] Rundo JV. Obstructive sleep apnea basics. Cleve Clin J Med. 2019;86(9 Suppl 1):2–9. doi: 10.3949/ccjm.86.s1.02 31509498

[2] White DP. Sleep-related breathing disorder.2. Pathophysiology of obstructive sleep apnoea. Thorax. 1995;50(7):797–804. doi: 10.1136/thx.50.7.797 7570420 PMC474658

[3] Redline S, Yenokyan G, Gottlieb DJ, Shahar E, O’Connor GT, Resnick HE, et al. Obstructive sleep apnea-hypopnea and incident stroke: the sleep heart health study. Am J Respir Crit Care Med. 2010;182(2):269–277. doi: 10.1164/rccm.200911-1746OC 20339144 PMC2913239

[4] Peker Y, Hedner J, Norum J, Kraiczi H, Carlson J. Increased incidence of cardiovascular disease in middle-aged men with obstructive sleep apnea: a 7-year follow-up. Am J Respir Crit Care Med. 2002;166(2):159–165. doi: 10.1164/rccm.2105124 12119227

[5] Peppard PE, Szklo-Coxe M, Hla KM, Young T. Longitudinal association of sleep-related breathing disorder and depression. Arch Intern Med. 2006;166(16):1709–1715. doi: 10.1001/archinte.166.16.1709 16983048

[6] Kendzerska T, Gershon AS, Hawker G, Tomlinson G, Leung RS. Obstructive sleep apnea and incident diabetes. A historical cohort study. Am J Respir Crit Care Med. 2014;190(2):218–225. doi: 10.1164/rccm.201312-2209OC 24897551

[7] Arita A, Sasanabe R, Hasegawa R, Nomura A, Hori R, Mano M, et al. Risk factors for automobile accidents caused by falling asleep while driving in obstructive sleep apnea syndrome. Sleep Breath. 2015;19(4):1229–1234. doi: 10.1007/s11325-015-1145-7 25716746 PMC4662952

[8] Maeder MT, Schoch OD, Rickli H. A clinical approach to obstructive sleep apnea as a risk factor for cardiovascular disease. Vasc Health Risk Manag. 2016;12:85–103. doi: 10.2147/VHRM.S74703 27051291 PMC4807890

[9] Pollicina I, Maniaci A, Lechien JR, Iannella G, Vicini C, Cammaroto G, et al. Neurocognitive Performance Improvement after Obstructive Sleep Apnea Treatment: State of the Art. Behav Sci (Basel). 2021;11(12). doi: 10.3390/bs11120180 34940115 PMC8698492

[10] Canessa N, Castronovo V, Cappa SF, Aloia MS, Marelli S, Falini A, et al. Obstructive sleep apnea: brain structural changes and neurocognitive function before and after treatment. Am J Respir Crit Care Med. 2011;183(10):1419–1426. doi: 10.1164/rccm.201005-0693OC 21037021

[11] Certal VF, Pratas R, Guimarães L, Lugo R, Tsou Y, Camacho M, et al. Awake examination versus DISE for surgical decision making in patients with OSA: A systematic review. Laryngoscope. 2016;126(3):768–774. doi: 10.1002/lary.25722 26484801

[12] Hou H, Zhao Y, Yu W, Dong H, Xue X, Ding J, et al. Association of obstructive sleep apnea with hypertension: A systematic review and meta-analysis. J Glob Health. 2018;8(1):010405. doi: 10.7189/jogh.08.010405 29497502 PMC5825975

[13] Olaithe M, Bucks RS, Hillman DR, Eastwood PR. Cognitive deficits in obstructive sleep apnea: Insights from a meta-review and comparison with deficits observed in COPD, insomnia, and sleep deprivation. Sleep Med Rev. 2018;38:39–49. doi: 10.1016/j.smrv.2017.03.005 28760549

[14] Attal P, Chanson P. Endocrine aspects of obstructive sleep apnea. J Clin Endocrinol Metab. 2010;95(2):483–495. doi: 10.1210/jc.2009-1912 20061419

[15] Reutrakul S, Mokhlesi B. Obstructive Sleep Apnea and Diabetes: A State of the Art Review. Chest. 2017;152(5):1070–1086. doi: 10.1016/j.chest.2017.05.009 28527878 PMC5812754

[16] Pinto JA, Ribeiro DK, Cavallini AF, Duarte C, Freitas GS. Comorbidities Associated with Obstructive Sleep Apnea: a Retrospective Study. Int Arch Otorhinolaryngol. 2016;20(2):145–150. doi: 10.1055/s-0036-1579546 27096019 PMC4835326

[17] Gupta MA, Simpson FC. Obstructive sleep apnea and psychiatric disorders: a systematic review. J Clin Sleep Med. 2015;11(2):165–175. doi: 10.5664/jcsm.4466 25406268 PMC4298774

[18] Baty F, Putora PM, Isenring B, Blum T, Brutsche M. Comorbidities and burden of COPD: a population based case-control study. PLoS One. 2013;8(5):e63285. doi: 10.1371/journal.pone.0063285 23691009 PMC3656944

[19] Skorin L, Knutson R. Ophthalmic Diseases in Patients With Obstructive Sleep Apnea. J Am Osteopath Assoc. 2016;116(8):522–529. 27455101 10.7556/jaoa.2016.105

[20] Lin CH, Perger E, Lyons OD. Obstructive sleep apnea and chronic kidney disease. Curr Opin Pulm Med. 2018;24(6):549–554. doi: 10.1097/MCP.0000000000000525 30239379

[21] Chou TC, Liang WM, Wang CB, Wu TN, Hang LW. Obstructive sleep apnea is associated with liver disease: a population-based cohort study. Sleep Med. 2015;16(8):955–960. doi: 10.1016/j.sleep.2015.02.542 26116463

[22] Strausz S, Kiiskinen T, Broberg M, Ruotsalainen S, Koskela J, Bachour A, et al. Sleep apnoea is a risk factor for severe COVID-19. BMJ Open Respir Res. 2021;8(1). doi: 10.1136/bmjresp-2020-000845 33436406 PMC7804843

[23] Pace A, Iannella G, Rossetti V, Visconti IC, Gulotta G, Cavaliere C, et al. Diagnosis of Obstructive Sleep Apnea in Patients with Allergic and Non-Allergic Rhinitis. Medicina (Kaunas). 2020;56(9). doi: 10.3390/medicina56090454 32911862 PMC7559128

[24] Lyons PG, Zadravecz FJ, Edelson DP, Mokhlesi B, Churpek MM. Obstructive sleep apnea and adverse outcomes in surgical and nonsurgical patients on the wards. J Hosp Med. 2015;10(9):592–598. doi: 10.1002/jhm.2404 26073058 PMC4560995

[25] Mohananey D, Villablanca PA, Gupta T, Agrawal S, Faulx M, Menon V, et al. Recognized Obstructive Sleep Apnea is Associated With Improved In-Hospital Outcomes After ST Elevation Myocardial Infarction. J Am Heart Assoc. 2017;6(7):e006133. doi: 10.1161/JAHA.117.006133 28729411 PMC5586313

[26] Bailly S, Galerneau LM, Ruckly S, Terzi N, Schwebel C, Dupuis C, et al. Impact of obstructive sleep apnea on ICU patient’s prognosis—insights from a French ICU cohort. European Respiratory Journal. 2019;54(suppl 63). doi: 10.1183/13993003.congress-2019.PA866

[27] Ismailov E, Rose-Reneau Z, Arellanes R, Dang A, Schirmer D. The impact of OSA on the outcomes of patients admitted with a myocardial infarction. Chest. 2019;156(4):A355. doi: 10.1016/j.chest.2019.08.397

[28] Lindenauer PK, Stefan MS, Johnson KG, Priya A, Pekow PS, Rothberg MB. Prevalence, treatment, and outcomes associated with OSA among patients hospitalized with pneumonia. Chest. 2014;145(5):1032–1038. doi: 10.1378/chest.13-1544 24371839 PMC4011652

[29] Joshi AA, Hajjali RH, Gokhale AV, Smith T, Dey AK, Dahiya G, et al. Outcomes of patients hospitalized for acute pulmonary embolism by obstructive sleep apnea status. Pulm Circ. 2021;11(2):2045894021996224. doi: 10.1177/2045894021996224 33854766 PMC8013707

[30] von Elm E, Altman DG, Egger M, Pocock SJ, Gøtzsche PC, Vandenbroucke JP. The Strengthening the Reporting of Observational Studies in Epidemiology (STROBE) statement: guidelines for reporting observational studies. PLoS Med. 2007;4(10):e296. doi: 10.1371/journal.pmed.0040296 17941714 PMC2020495

[31] Iannella G, Maniaci A, Magliulo G, Cocuzza S, La Mantia I, Cammaroto G, et al. Current challenges in the diagnosis and treatment of obstructive sleep apnea syndrome in the elderly. Pol Arch Intern Med. 2020;130(7-8):649–654. 32250579 10.20452/pamw.15283

[32] May AM, Van Wagoner DR, Mehra R. OSA and Cardiac Arrhythmogenesis: Mechanistic Insights. Chest. 2017;151(1):225–241. doi: 10.1016/j.chest.2016.09.014 27693594 PMC5989643

[33] Di Mauro P, Cocuzza S, Maniaci A, Ferlito S, Rasà D, Anzivino R, et al. The Effect of Adenotonsillectomy on Children’s Behavior and Cognitive Performance with Obstructive Sleep Apnea Syndrome: State of the Art. Children (Basel). 2021;8(10). doi: 10.3390/children8100921 34682186 PMC8535044

[34] Bolona E, Hahn PY, Afessa B. Intensive care unit and hospital mortality in patients with obstructive sleep apnea. J Crit Care. 2015;30(1):178–180. doi: 10.1016/j.jcrc.2014.10.001 25457113

[35] Taweesedt P, Djurdjevic N, Pattharanitima P, Ali F, Kim JW. Mortality in patients with or without OSA who required invasive mechanical ventilation: a propensity score-matched analysis. Chest. 2020;158(4):A2339. doi: 10.12659/AJCR.923266 32513908 PMC7304654

[36] Lin P, Li X, Zhang J, Liang Z. Association Between Obstructive Sleep Apnea and Reduced Mortality in Critically Ill Patients: A Propensity Score-Based Analysis. Int J Gen Med. 2021;14:4723–4729. doi: 10.2147/IJGM.S330752 34456584 PMC8387641

[37] Mokhlesi B, Hovda MD, Vekhter B, Arora VM, Chung F, Meltzer DO. Sleep-disordered breathing and postoperative outcomes after elective surgery: analysis of the nationwide inpatient sample. Chest. 2013;144(3):903–914. doi: 10.1378/chest.12-2905 23538745 PMC3760743

[38] Lavie P, Lavie L. Unexpected survival advantage in elderly people with moderate sleep apnoea. J Sleep Res. 2009;18(4):397–403. doi: 10.1111/j.1365-2869.2009.00754.x 19663998

[39] Chaudhary D, Khan A, Gupta M, Hu Y, Li J, Abedi V, et al. Obesity and mortality after the first ischemic stroke: Is obesity paradox real? PLoS One. 2021;16(2):e0246877. doi: 10.1371/journal.pone.0246877 33566870 PMC7875337

[40] Gupta T, Kolte D, Mohananey D, Khera S, Goel K, Mondal P, et al. Relation of Obesity to Survival After In-Hospital Cardiac Arrest. Am J Cardiol. 2016;118(5):662–667. doi: 10.1016/j.amjcard.2016.06.019 27381664

[41] Sforza E, Roche F. Chronic intermittent hypoxia and obstructive sleep apnea: an experimental and clinical approach. Hypoxia (Auckl). 2016;4:99–108. doi: 10.2147/HP.S103091 27800512 PMC5085272

[42] Dale EA, Ben Mabrouk F, Mitchell GS. Unexpected benefits of intermittent hypoxia: enhanced respiratory and nonrespiratory motor function. Physiology (Bethesda). 2014;29(1):39–48. doi: 10.1152/physiol.00012.2013 24382870 PMC4073945

[43] Alejos D, Festic E, Guru P, Moss JE. Neurological outcomes of patients with history of obstructive sleep apnea after a cardiac arrest. Resuscitation. 2017;119:13–17. doi: 10.1016/j.resuscitation.2017.07.027 28764949

[44] Festic N, Alejos D, Bansal V, Mooney L, Fredrickson PA, Castillo PR, et al. Sleep Apnea in Patients Hospitalized With Acute Ischemic Stroke: Underrecognition and Associated Clinical Outcomes. J Clin Sleep Med. 2018;14(1):75–80. doi: 10.5664/jcsm.6884 29198297 PMC5734897

[45] Lavie L. Oxidative stress in obstructive sleep apnea and intermittent hypoxia–revisited–the bad ugly and good: implications to the heart and brain. Sleep Med Rev. 2015;20:27–45. doi: 10.1016/j.smrv.2014.07.003 25155182

[46] Shah N, Redline S, Yaggi HK, Wu R, Zhao CG, Ostfeld R, et al. Obstructive sleep apnea and acute myocardial infarction severity: ischemic preconditioning? Sleep Breath. 2013;17(2):819–826. doi: 10.1007/s11325-012-0770-7 23090861

[47] Ludka O, Stepanova R, Sert-Kuniyoshi F, Spinar J, Somers VK, Kara T. Differential likelihood of NSTEMI vs STEMI in patients with sleep apnea. Int J Cardiol. 2017;248:64–68. doi: 10.1016/j.ijcard.2017.06.034 28720312

[48] Ozeke O, Ozer C, Gungor M, Celenk MK, Dincer H, Ilicin G. Chronic intermittent hypoxia caused by obstructive sleep apnea may play an important role in explaining the morbidity-mortality paradox of obesity. Med Hypotheses. 2011;76(1):61–63. doi: 10.1016/j.mehy.2010.08.030 20822856

